# Draft Genome Sequence of the Piezotolerant and Crude Oil-Degrading Bacterium *Rhodococcus qingshengii* Strain TUHH-12

**DOI:** 10.1128/genomeA.00268-15

**Published:** 2015-04-09

**Authors:** Sara A. Lincoln, Trinity L. Hamilton, Ana Gabriela Valladares Juárez, Martina Schedler, Jennifer L. Macalady, Rudolf Müller, Katherine H. Freeman

**Affiliations:** a Department of Geosciences, Pennsylvania State University, University Park, Pennsylvania, USA; b Institute of Technical Biocatalysis, Hamburg University of Technology, Hamburg, Germany

## Abstract

We report here the draft genome sequence of *Rhodococcus qingshengii* strain TUHH-12. The ability of this piezotolerant bacterium to grow on crude oil and tetracosane as sole carbon sources at 150 × 10^5^ Pa makes it useful in studies of hydrocarbon degradation under simulated deep-sea conditions.

## GENOME ANNOUNCEMENT

The 2010 Deepwater Horizon oil spill and the recent expansion of deep-sea drilling highlight the importance of understanding biodegradation processes that control the long-term fate of hydrocarbons in deep marine sediments. Although high pressure can inhibit the growth of sedimentary hydrocarbon-degrading bacteria (1), laboratory biodegradation studies are typically performed at ambient pressure, and the extent to which their results are applicable to the deep ocean is uncertain. Comparative studies of piezotolerant hydrocarbon-degrading bacteria grown at surface and deep-sea pressures may help identify the biochemical mechanisms responsible for pressure-related inhibition.

We report here the draft genome sequence of *Rhodococcus qingshengii* strain TUHH-12, which we have used in high-pressure hydrocarbon degradation studies (2). This strain was isolated from water underlying sea ice in Spitsbergen, Norway, by aerobic enrichment at 4°C in mineral medium supplemented with crude oil and decane. The recent isolation of another *R. qingshengii* strain (GenBank accession no. KC894023.1) from marine sediment suggests that the results of experiments using *R. qingshengii* strain TUHH-12 are relevant for deep-sea biodegradation studies.

We sequenced 100 ng of genomic DNA extracted from *R. qingshengii* TUHH-12 using an Ion PGM system (Life Technologies, Carlsbad, CA), generating 1,319,688 reads, with an *N*_50_ of 226 bp, an average length of 205 bp, and a G+C content of 61.7%. The coverage was approximately 35×. The reads were assembled using SPAdes (version 3.0.0 [3]), resulting in 349 contigs, with a total sequence length of 7,430,810 bp and an *N*_50_ of 133,054 bp. The longest contig was 418,804 bp. The contigs were annotated using RAST (4).

Rhodococci are agents of contaminant biodegradation in diverse environments. Their large genomes enable catabolic versatility, as alkanes, polycyclic, and halogenated aromatic hydrocarbons, as well as ether bond-containing compounds, such as ethyl tert-butyl ether (ETBE), can all serve as carbon substrates for *Rhodococcus* species (5).

The *R. qingshengii* TUHH-12 genome encodes multiple alkane-1 monooxygenases and putative cytochrome P450 hydroxylases, which are components of aerobic alkane degradation pathways. Enzymes in the homogentisate pathway of aromatic compound degradation that are present in the TUHH-12 genome include homogentisate 1,2-dioxygenase, fumarylacetoacetase, 4-hydroxyphenylpyruvate dioxygenase, and IclR family transcriptional regulators. Dehalogenation pathways are represented by haloalkane and type II haloacid dehalogenases. A putative ETBE degradation gene is present. The TUHH-12 genome also encodes probable dibenzothiophene desulfurization pathway enzymes, including multiple copies of dibenzothiophene desulfurization enzyme B.

The capacity for exopolysaccharide (EPS) biosynthesis by TUHH-12 is indicated in the genome by the presence of glycosyl transferase, galactose phosphotransferase, undecaprenyl-phosphate galactose phosphotransferase, GDP-l-fucose synthetase, and other genes associated with capsular EPS production. EPS is thought to enhance hydrocarbon solubilization and floc formation by rhodococci (6) and other hydrocarbon-degrading bacteria, and it likely promoted the formation of oil-rich aggregates in the Gulf of Mexico following the Deepwater Horizon oil spill (7).

Comparative transcriptomic studies of *R. qingshengii* TUHH-12 grown under ambient laboratory and simulated seafloor pressure conditions will likely provide new insights into hydrocarbon degradation in the deep sea.

### Nucleotide sequence accession numbers.

This whole-genome shotgun project has been deposited at DDBJ/EMBL/GenBank under the accession no. JNCU00000000, and the version described here is the first. The raw reads were deposited in the NCBI Sequence Read Archive under the accession no. SRX530906. *R. qingshengii* strain TUHH-12 was deposited in the DSMZ culture collection under the accession no. DSM 46766.
